# Non-surgical interventions for proliferative vitreoretinopathy—a systematic review

**DOI:** 10.1038/s41433-025-03846-7

**Published:** 2025-05-28

**Authors:** Guy Hunter, Nairn Maclean, Stephanie Watson, Marcus Colyer, James Leong, Rupesh Agrawal, Scott F. McClellan, Kyle E. Miller, Fasika A. Woreta, Matthew C. Caldwell, Tom Williamson, William Gensheimer, Kara Cavuoto, Annette K. Hoskin, Andrés Rousselot Ascarza, William G. Felipe Dhawahir-Scala, Gangadhara Sundar, Robert A. Mazzoli, Peter Shah, Malcolm Woodcock, Ferenc Kuhn, Renata SM Gomes, Grant A. Justin, Richard J. Blanch

**Affiliations:** 1 Institute of Naval Medicine, Gosport, UK; 2 Army Medical Services, Camberley, UK; 3 Save Sight Institute, The University of Sydney, Sydney, NSW Australia; 4 Uniformed Services University of the Health Sciences, Bethesda, MD USA; 5 Sydney Eye Hospital, Sydney, NSW Australia; 6 National Healthcare Group Eye Institute, Tan Tock Seng Hospital, Singapore, Singapore; 7 Singapore Eye Research Institute, Singapore, Singapore; 8 Lee Kong Chian School of Medicine, Singapore, Singapore; 9 Duke NUS Medical School, Singapore, Singapore; 10 Vision Center of Excellence, Research & Development Directorate (J-9), Defence Health Agency, Baltimore, MD USA; 11 HQ Army Air Corps, Middle Wallop, UK; 12 Department of Ophthalmology, Navy Medical Center Portsmouth, Portsmouth, VA USA; 13 Wilmer Eye Institute, Johns Hopkins University School of Medicine, Baltimore, MD USA; 14 Department of Ophthalmology, San Antonio Uniformed Services Health Education Consortium, San Antonio, TX USA; 15 Department of Engineering and Biological Sciences, University of Surrey, Surrey, UK; 16 Department of Ophthalmology, St Thomas’ Hospital, London, UK; 17 White River Junction Veterans Administration Medical Center, White River Junction, VT USA; 18 Dartmouth-Hitchcock Medical Center, Lebanon, NH USA; 19 Bascom Palmer Eye Institute, University of Miami, Miami, FL USA; 20 Lions Eye Institute, University of Western Australia, Perth, WA Australia; 21 Consultorios Oftalmológicos Benisek-Ascarza, Ciudad Autónoma de Buenos Aires, Buenos Aires, Argentina; 22 Manchester Royal Eye Hospital, Manchester, UK; 23 Department of Ophthalmology, National University Hospital, Singapore, Singapore; 24 Birmingham Institute for Glaucoma Research, Birmingham, UK; 25 Ophthalmology Department, University Hospitals Birmingham NHS Foundation Trust, Birmingham, UK; 26 Worcestershire Acute Hospitals NHS Trust, Worcester, UK; 27 Helen Keller Eye Research Foundation, Birmingham, AL USA; 28 Northern Hub for Veterans and Military Families Research, Northumbria University, Newcastle, UK; 29 Duke Eye Center, Duke University Hospitals, Durham, NC USA; 30 Academic Department of Military Surgery and Trauma, Royal Centre for Defence Medicine, Birmingham, UK; 31 Neuroscience and Ophthalmology, School of Infection, Inflammation, and Immunology, University of Birmingham, Birmingham, UK

**Keywords:** Drug therapy, Retinal diseases, Trauma, Inflammation

## Abstract

Proliferative Vitreoretinopathy (PVR) is the most common cause of surgical failure after retinal detachment (RD) repair, complicating up to 10% of spontaneous RD repairs and 50% of open globe injury-related RD. Early surgical intervention is currently the only intervention that reduces PVR incidence. An effective non-surgical intervention would be valuable in reducing PVR incidence and/or severity, particularly where access to surgery is limited or may be delayed. To define the evidence base for non-surgical management options to prevent and treat Proliferative Vitreoretinopathy (PVR) in retinal detachment and trauma, we searched PubMed, Clinicaltrials.gov, Medline and CINAHL for randomised controlled trials (RCTs) of non-surgical interventions to prevent or treat established PVR with no restriction on language or start date up until November 2024. All non-surgical interventions were considered with no restrictions. We considered outcomes of post-operative PVR (including retinal reattachment rate) and visual acuity and performed Risk of Bias (RoB) assessments using the Cochrane RoB2 tool. We identified 27 papers which included 1981 patients in studies of primary prevention and 1394 patients in studies of treatment of established PVR. While several studies of various agents individually demonstrated some improvements, the reviewers found concerns with RoB and the results were not replicated in the larger included studies. Multiple studies have investigated non-surgical interventions for PVR after RRD repair and trauma, but none have yet demonstrated clinically significant, repeatable benefits. Improved understanding of PVR pathobiology, along with larger prospective studies of existing preventative strategies may lead to the development of newer and more effective interventions.

## Introduction

Proliferative vitreoretinopathy (PVR) is a multifactorial fibroproliferative process involving an abnormal wound healing response characterised by the growth and contraction of cellular membranes within the vitreous cavity and on both sides of the retinal surface as well as intraretinal fibrosis [1]. PVR becomes clinically significant by causing progressive retinal detachment, reopening treated retinal breaks, creating new retinal breaks, or distorting the macula. It is common in cases of open globe injury (OGI) and is the most common cause for surgical failure after rhegmatogenous retinal detachment (RRD) repair. The incidence of PVR after repair of atraumatic RRD is 5–10% [2], and has remained largely unchanged in prospective studies despite the evolution of vitreoretinal techniques and improved understanding of clinical risk factors over the past 25 years [3]. One study reported that RRD occurred in 29% (26%–32% CI) of open globe injury (OGI) cases, of which 27% detached in the first 24 h, 47% within 1 week, and 72% within one month [4]. All such cases are at high risk of subsequently developing PVR, as demonstrated in other studies where PVR was already present in over 50% of ocular trauma cases who underwent vitrectomy within 10 days of injury [5, 6].

After RRD, the inflammatory ‘PVR cascade’ occurs with multiple steps, each representing potential therapeutic targets for pharmacological intervention. These include: retinal ischaemia with associated breakdown of the blood-retinal barriers, photoreceptor cell death and its associated inflammation, and retinal pigment epithelial (RPE) cell and retinal glial cell migration to the vitreous and inner retinal surfaces associated with the physical effects of trauma [1, 3, 7]. Blood-retinal barrier disruption, combined with retinal apoptosis, causes an increase in vitreous chemotactic and mitogenic factors [8], with influx of cytokines and growth factors from the systemic circulation and local production by infiltrating inflammatory cells that interact with retinal and RPE cells to further increase vitreous cytokine production [9]. Subsequent RPE epithelial-mesenchymal transition (EMT) to a fibroblastic phenotype generates cells with greater migratory capacity, invasiveness, contractability, resistance to apoptosis, and extracellular matrix production [10].

Severity of PVR is assessed and classified in clinical practice and research based on the ophthalmoscopic appearance, the subjective evaluation of the amount of membrane contraction and its distribution [11]. The initial classification by the Retina Society Terminology Committee subdivided PVR into four stages A, B, C and D from minimal to massive PVR [2], later modified in the updated classification to grades A, B and C, with Grade A being vitreous cells or haze, Grade B describing subclinical contraction or wrinkling and Grade C divided into Ca, anterior to the equator, and Cb, posterior to the equator [12]. Clinical outcomes may be assessed by retinal attachment status, visual acuity or by other clinical signs which act as markers or sequelae of PVR activity such as vitreous haze or epiretinal membrane formation.

PVR development involves a sequence of: retinal ischaemia, occurring immediately after detachment; photoreceptor apoptosis; and formation and subsequent contraction of fibrotic membranes [13]. Both ischaemia, apoptosis and the primary injury or retinal tear are associated with the release of pro-inflammatory, fibrogenic and mitogenic signalling molecules [14], which influence retinal pigment epithelial cells to proliferate and undergo epithelial-mesenchymal transition into fibroblasts and deposit collagen and ECM [13, 15, 16].

Pharmacologic interventions have targeted inflammation, cell proliferation, and fibrosis pathways, for instance: corticosteroid therapy targets broad inflammatory pathways; anti-VEGF therapies may be anti-inflammatory by inhibition of immune cell chemotaxis [17]; heparin modulates pro-fibrotic growth factors actions including FGF and VEGF [18]; and the various anti-metabolite and related drugs such as 5-fluorouracil (5-FU) and daunorubicin inhibit cell proliferation, and therefore fibroblast and immune cell proliferation. Nonetheless, PVR management remains challenging with no therapies in current clinical practice to prevent or reverse the disease process [3].

At present, there is only evidence to support vitrectomy (and its related procedures such as washout of vitreous haemorrhage, removal of established PVR membranes, retinopexy, and retinectomy) as an effective management for PVR [19]. A recent paper also suggests that prophylactic chorioretinectomy as part of vitrectomy may be effective in PVR prevention [20]. We will therefore attempt to define the evidence for non-surgical interventions in the primary prevention and treatment (including prevention/reduction of recurrence after surgery) of PVR after OGI and RRD which may mitigate the sight-threatening effects of PVR in situations where rapid access to vitrectomy may not be possible, such as in trauma patients who are temporarily unfit for general anaesthesia, in rural/austere environments or in conflict zones with prolonged casualty evacuation timelines.

This was accomplished by performing a systematic review of randomised controlled trial (RCT) data on non-surgical PVR treatments published in peer-reviewed journals up to the start of the review process in November 2024. The review was registered on PROSPERO (CRD42024610938).

## Methods

This systematic review was conducted in accordance with the PRISMA (Preferred Reporting Items for Systematic Reviews and Meta-Analyses) statement [21].

### Inclusion and exclusion criteria

Studies included were RCTs in any language or country of origin, published in an indexed medical journal up to November 2024. These studies evaluated interventions aiming either to prevent PVR in patients who had suffered OGI or RRD and undergone vitrectomy (primary prevention), or interventions used as an adjunct to improve visual outcomes or reduce PVR recurrence in patients who are undergoing vitrectomy for already established PVR (treatment). Exclusion criteria were non-RCT studies and studies of proliferative diabetic retinopathy.

### Search strategy

Four scientific databases (PubMed, Clinicaltrials.gov, Medline and CINAHL) were searched using the search strings defined in the updated protocol including the MeSH term “vitreoretinopathy, proliferative” and others OR keyword PVR, as defined in the PROSPERO registration (CRD42024610938). Results were restricted to RCT as previously described [22]. The search strategy was changed from the original protocol in order to better capture the intended data. Two independent reviewers (GH and RJB) individually reviewed all titles from the initial searches, duplicates were eliminated and papers selected for full text review on the basis of the titles and abstracts using the Rayyan online tool for comparison [23]. Decisions on inclusion were made on the basis of full text review and disagreements resolved by discussion.

### Data extraction

Data were extracted by two reviewers (GH and NM) working independently. Data extracted included intervention type, population targeted, primary and secondary outcomes as reported and any safety concerns. The primary outcomes that we aimed to report were anatomic success of retinal reattachment and visual acuity, without limit on duration of follow up.

### Risk of bias

Risk of Bias assessment was carried independently out by two authors (GH, RJB) using the RoB2 tool [24], with disagreements resolved by discussion.

## Results

### Included studies

**Fig. 1:**
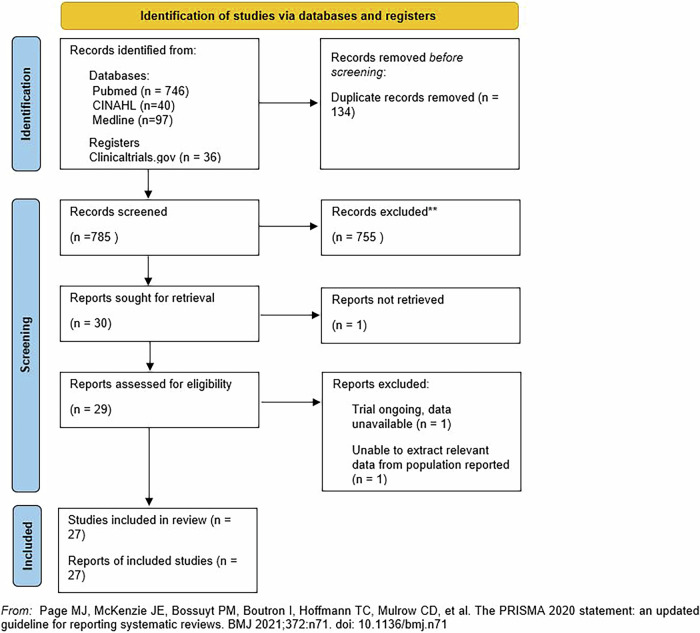
PRISMA Flow Diagram. Details of paper selection process.

Twenty-seven studies [25–52] met the inclusion criteria and were included in the review, which together included 3375 patients recruited to RCTs, separated into those aimed at the primary prevention of PVR after RRD or OGI (*n* = 1981) and the secondary treatment of PVR recurrence (*n* = 1394). These totals include numbers from one study, which included both primary prevention of PVR (*n* = 45) and secondary treatment (*n* = 45) [37]. A Preferred Reporting Items for Systematic Reviews and Meta-Analyses (PRISMA) flow diagram (Fig. 1) demonstrates the study selection process.

### Characteristics of included studies

Patient and study characteristics in the included studies are presented in Table 1. The mean age of included patients ranged from 9.6 to 68.53 years, however, only one paper [44] studied a population where >5% of the patients were under 18 years old. 68.3% were male (where gender information was available) and inclusion criteria varied from primary RRD to severe detachments with established PVR, both with and without a history of trauma. Some studies excluded patients for a wide range of criteria, normally related to other inflammatory processes such as autoimmune disease, diabetic retinopathy, glaucoma, chronic uveitis, or complex retinal detachments such as giant retinal tears.

**Table 1:** Characteristics of included studies.

Intervention	Author	Date/Country	Age	Gender	Study design	Inclusion criteria	Exclusion criteria
Anti-Inflammatory							
Acetyl-salicylic acid	Kralinger et al.	2010, Austria	Intervention: 61.1 ± 6.3, control: 69 ± 15.7	M = 15, F = 14	Multi-center double-masked prospective randomised controlled study	PVR at least grade C and visual acuity of less than 20/40	Under 19 years old; pregnancy; allergy to aspirin; uncontroled hypertension with BP > 150/100; cataract; uveitis; vascular retinopathy
Colchicine	Ahmadieh et al.	2015, Iran	No data	No data	Double masked randomised placebo-controlled trial	RRD undergoing scleral buckling for primary repair	Nil specified
Corticosteroids	Ahmadieh, Feghhi et al.	2008, Iran	Intervention: 54.5 ± 18.8. Control 45.7 ± 20.7. *P* = 0.07	M = 59, F = 16	Prospective randomised controlled trial	Eyes undergoing primary vitrectomy for rhegmatogenous retinal detachment complicated by PVR grade C or eyes with previous vitrectomy, scleral buckling, or both with recurrent retinal detachment resulting from PVR	Diabetic retinopathy; history of penetrating trauma; giant retinal tears; chronic uveitis; previous intraocular injection of steroids
Corticosteroids	Trenado-Luna et al.	2023, Mexico	Intervention: 55.5 ± 14.84 years. Control 50.15 ± 18.35 years	M = 22, F = 16	Prospective randomised controlled trial	Patients undergoing PPV for PVR Grade B or C	Open globe trauma; definitive glaucoma with high risk of visual damage due to IOP rise; known steroid IOP response; pregnant or breastfeeding; suspected infection; reported reactions/allergies to prior dexamethasone implantation; intraoperative lensectomy; anterior chamber lens implant; history of immunodeficiency; Cushing’s syndrome or uncontrolled/newly diagnosed autoimmune disorders; incomplete information in ophthalmological notes/follow up. There were no restrictions on the number of previous surgeries.
Corticosteroids	Banerjee et al.	2017, UK	Intervention: 60.6 ± 14.3 years. Control: 61.6 ± 13.9 years.	M = 86, F = 54	Phase IIIb single-center participant-masked prospective randomised controlled clinical trial	Patients undergoing PPV with silicone oil for established grade C PVR.	Open globe injury; a diagnosis of ocular hypertension on 2 or more pressure lowering medications or a definite diagnosis of glaucoma (if in the opinion of a glaucoma specialist, the patient is at high risk of visual damage from increased intraocular pressure [IOP]); uncontrolled uveitis; previous steroid-induced glaucoma; proliferative diabetic retinopathy or vasculopathy; pregnant or breastfeeding females; previous known adverse reaction to Ozurdex (Allergan Inc, Irvine, CA); suspected ocular/periocular infection (e.g., herpes simples virus, varicella zoster virus, mycobacterial infection, fungal disease); aphakia or patients in whom a lensectomy is planned at time of surgery; and preexisting anterior chamber intraocular lens. There were no restrictions on the number of previous vitreoretinal procedures.
Corticosteroids	Casswell et al.	2023, UK	Intervention: 46.8 ± 17.3 years. Control: 42.7 ± 15.3 years.	M = 246, F = 34	Phase 3 multicenter randomised controlled clinical trial	Over age 18, full thickness open globe trauma, able to consent and attend follow-up for 6 months	Age under 18 years old; pre-existing uncontrolled uveitis; previous diagnosis of steroid-induced glaucoma; pregnant or breast-feeding women; allergy or previous reaction to TA; inability to attend follow-up; inability to give written consent; current or planned systemic corticosteroid use of a dose above physiological levels (>10 mg prednisolone). The indication for vitrectomy following OGI was at the discretion of the operating surgeon.
Corticosteroids	Banerjee, Xing et al.	2016, UK	Intervention: 44 (±16), Control: 37 (±13)	M = 34, F = 6	Randomised controlled clinical trial	Open globe injury undergoing pars plana vitrectomy (PPV) either following primary injury repair or as a primary procedure itself, intraocular foreign body (IOFB) injuries.	History of (a) glaucoma, (b) PPV surgery to the affected eye, (c) known adverse reaction to any of the IMPs; pregnant or breastfeeding females; enrolment in other clinical trials; inability to attend regular follow-up; unable to give written informed consent
Corticosteroids	Koerner et al.	2012, Germany	Intervention: 54.5 ± 13.8, Control: 54.1 ± 14.4	M = 144, F = 76	Prospective randomised double-blind placebo controlled trial	Adult patients age 18-75 hospitalised for treatment of RRD between January 1994 and April 1999.	Preoperative PVR Grade C; previous vitreoretinal surgery; uveitis; glaucoma; pregnancy; systemic diseases such as diabetes; arterial hypertension; peptic ulcer; immune deficiency
Corticosteroids	Dehghan et al.	2010, Iran	Intervention: 48 (±14). Control: 42 (±17). *p* = 0.12	M = 34, F = 18	Double-masked randomised placebo-controlled trial	Phakic eyes with acute RRD and PVR grade A or B.	Aphakia; pseudophakia; diabetes; longstanding retinal detachment; history of vitreoretinal surgery; PVR grade C; myopia more than 6.00 D; macular degeneration; macular hole; history of long-term steroid use; contraindications for oral steroids administration. Interoperative complications: haemorrhage from sclerotomy site; retinal or vetrious incerceration; patients requiring intravetrial injection of fluid, gas or air.
Corticosteroids	Guo et al.	2023, China	Intervention: 47.67 ± 12.26, Control: 50.73 ± 11.83	M = 58, F = 10	Single-center participant-masked prospective randomised placebo-controlled clinical trial	Age 18 years or older, globe rupture whose wound involved zone III, emergency ocular repair surgery completed within 24 h post injury, and patients who voluntarily underwent vitreoretinal surgery	Younger than 18 years; diagnosis of penetrating or perforating injury, traumatic endophthalmitis, IOFB, fracture of the optic nerve canal; congenital anomalies of the eye; history of ocular trauma, intraocular surgery; periocular infectious disease; adverse reaction to TA; past or present use of high-dose glucocorticoids; pregnant or lactating women; history of psychiatric disease
Anti-metabolite/Anti-proliferative							
Daunorubicin	Kumar et al.	2002, India	Intervention: 52.4 ± 7.6, Control: 54.3 ± 8.7	M = 27, F = 3	Prospective randomised controlled trial	18 years or older and a primary rhegmatogenous retinal detachment with a PVR of stage D1 or more severe according to the Retina Society classification	Glaucoma; uveitis; diabetic retinopathy; previous ocular trauma; prior vitrectomy; receiving corticosteroid/immunosuppressive therapy
Daunorubicin	Wiedemann et al.	1998, Germany	Intervention: 62.9 ± 12.5 years, Control: 61.1 ± 13.0 years	M = 161, F = 125	Multicenter prospective randomised controlled trial	Age of 18 years or older, PVR after rhegmatogenous retinal detachment, preoperative biomicroscopic staging of C2 or more advanced, with probable use of silicone oil, and written informed consent. One prior vitrectomy without silicone oil tamponade, retinotomy, and intraocular medication was accepted.	Diabetic retinopathy; glaucoma with visual field defect; uveitis with posterior segment involvement; previous ocular trauma; previous giant tears; preoperative attached macula; systemic cytostatic therapy or radiation; pregnancy.
DNA RNA chimeric ribozyme	Schiff et al.	2007, USA	Intervention High: 59.4 years, Intervention Low: 58.6 years, Control: 62.1 years [mean values]	M = 116, F = 59	Multicenter double-masked prospective randomised placebo-controlled trial	Presence of primary or recurrent RD with grade C or worse PVR (Retina Society classification), defined as the presence of at least 1 fixed retinal fold in at least 1 quadrant, in a patient at least 18 years of age with visual acuity of light perception or better.	Presence of severe nonproliferative or proliferative diabetic retinopathy or other preexisting ocular vasoproliferative diseases; a history of intraocular inflammatory disease; hereditary vitreoretinopathies.
5-FU and heparin	Charteris et al.	2004, UK	Intervention: 65.8 ± 14.77 year, Control: 66.2 ± 13.35 years	M = 97, F = 60	Double-masked prospective randomised placebo-controlled trial	Eyes with PVR grade C, Anterior or posterior with at least 1-clock hour involvement, types 1, 2, 4, or 5; males over 16 years of age; and postmenopausal females.	Giant retinal tears; posterior penitrating trauma; diabetic retinopathy; glaucoma; corneal opacity sufficiant to impair surgical view; no light perception vision; previous silicone oil injection; inability to give informed consent; inability to complete follow-up; unwillingness to accept randomisation.
5-FU and heparin	Wickham et al.	2007, UK	Intervention: 61.9 (SD 11.3), Control: 61.4 (SD 11.3)	M = 438, F = 203	Double-masked prospective randomised placebo-controlled trial	All RRDs treated with primary vitrectomy and Intraocular gas tamponade in males older than 16 and in postmenopausal women	RDDs treated with scleral buckle/silicone oil; giant retinal tears; penetrating trauma; diabetic retinopathy; glaucoma; corneal opacity; intended silicone oil tamponade; no light perception; unwilling to have randomisation; unable to give informed consent
5-FU and heparin	Ganekal et al.	2014 Saudi Arabia	Intervention:28.5 ± 6.8, Control: 38.5 ± 7.2	M = 31, F = 9	Double-masked prospective randomised placebo-controlled trial	Consecutive cases of RRD with grade B PVR or worse	Proliferative diabetic retinopathy; bleeding diathesis; hepatic or renal failure; glaucoma; giant tears; posterior penetrating trauma; corneal opacity; NLP vison; no valid consent; inability to accept randomisation.
5-FU and Heparin	Asaria et al.	2001, UK	Intervention: 62 (range 27–90) Control: 64.3 (range 18–93).	NI	Double-masked prospective randomised placebo-controlled trial	Patients >16 years old with RRD at risk of PVR	Proliferative diabetic retinopathy; posterior penetrating trauma; corneal opacity; premenopausal status; previous vitrectomy; consent issues; inability to follow-up; not accepting of randomisation
5-FU and heparin	Nasr et al	2024, Egypt	Intervention: 10.21 ± 3.39, Control: 9.0 ± 3.09	M = 27, F = 15	Prosepctive Randomised Controlled Trial	Children under 14 years of age with RRD undergoing primary repair were included, as well as those with preoperative PVR grade B or higher and those with high risk RRD: uveitis; large, giant, or multiple tears; vitreous haemorrhage; preoperative choroidal detachments; aphakia; and large detachments involving greater than two quadrants of the eye.	Children with RRD related to penetrating ocular trauma involving the posterior segment were excluded, as well as those with previous RD repair surgery, uncontrolled glaucoma or other concomitant ocular morbidities, patients with bleeding diathesis, hepatic and renal failure, corneal opacity sufficient to impair surgical view, no light perception vision or inability to complete follow-up.
5-FU and Heparin	Schaub et al.	2022, Germany	Intervention: 65 (58–70), Control: 63 (56–71)	M = 242, F = 83	Multicenter double-masked randomised controlled interventional trial with 1 interim analysis	Patients with primary RRD and high risk of PVR judged by flare photometry	Pre existing PVR grade C; inflammatory conditions affecting eye; previous trauma; giant retinal tears
Methotrexate	Rajan et al.	2024, India	Intervention 53.5 (38–67). Control 54.5 (19–71).	M = 32, F = 9	Randomised controlled trial	Over 18yo with RRD and PVR Grade C following RRD.	Previous retinal surgery, non-rhegmatogenous detachment, poor media clarity, monocular patients, pregnancy.
300 gy radiation	Binder et al.	1993, France and Austria	Intervention: 57.5 (47–68), control: 49.5 (41–58)	Intervention: M = 31, F = 32 (note that this must be a misprint as does not add up to 60)	Prospective randomised controlled study	Patients with RRD plus grade D1-3 PVR	Giant retinal tears; diabetic retinopathy; penetrating injury
Growth factor modulation							
Heparin	Williams et al.	1996, USA	Intervention: 14–82, control: 19–82	M = 35, F = 24	Prospective randomised controlled pilot study	Patients with RRD and severe PVR grade C3 or D	Diabetes; previous vitrectomy at the same institution
Heparin	Kumar, Nainiwal et al.	2003, India	Intervention: 44 ± 19.5, control: 51.27 ± 18.8	M = 21, F = 7	Prospective randomised controlled study	Patients > 18 years with RRD and severe PVR grade D1 - D3	Bleeding diatheses; proliferative diabetic retinopathy; liver and renal failure; under 18 years old; traumatic retinal detachment; failed previous surgery
Anti-VEGF	Tousi et al.	June 2016, Iran	53.7 ± 13.8	M = 17, F = 10	Randomised controlled pilot study	Primary RRD, PVR present (not worse than Grade B), suitable for PPV	Under 18 years old; previous retinal detachment surgery; trauma; uveitis; bleeding diathesis; hepatic/renal failure; diabetic retinopathy; AMD; GRT; macular hole
Other							
Infliximab	Younes et al.	2024, Egypt	Intervention: 51.1 (SD ± 13.3). Control 50.8 (SD ± 12.7)	M = 17, F = 43	Randomised Controlled Phase 2 Trial	Patients ≥ 18 years old with primary RRD and Grade C PVR	Patients with history of open globe iinjury, recurrent RRD or history of RRD surgery, laser retinopexy or cryopexy, retinal vascular diseases such as diabetic retinopathy, pregnant or breastfeeding females, inability to attend regular follow-up, or history of pulmonary or extra-pulmonary tuberculosis
Atropine and timolol	Tewari et al.	1994, India	Range of whole population 7–76	M = 62 F = 28	Double-masked prospective randomised controlled study	Failed primary RRD surgery in whom either PVR explained the faiure (PVR group) or in whom no visible retinal breaks explained it (non- PVR group)	Previous additional vitrectomy procedures; visible retinal holes; PVR worse than grade D1
Retinoic acid	Chang et al.	2008, Taiwan	Intervention: 57.4 ± 8.8, control: 52.0 ± 11.8	M = 25, F = 10	Prospective randomised controlled interventional case series	Primary RRD with PVR at least grade C	Giant retinal tears; penetrating trauma; RRD associated with systemic disease; diabetic retinopathy; pregnancy; age under 18; participation in other study; lost to follow-up

*BP* blood pressure, *M* male, *F* female, *IOP* intraocular pressure, *NLP* no light perception, *PVR* proliferative vitreoretinopathy, *RRD* rhegmatogenous retinal detachment, *TA* triamcinolone, *VEGF* vascular endothelial growth factor.

### Risk of bias of included studies

A risk of bias (RoB) assessment using the Cochrane RoB2 tool for each study is presented in Table 2. Concerns with reviewers relating to selection of reported results was the most common potential source of bias identified [26, 30, 32, 34, 35, 37, 39, 43, 45, 49–52], followed by deviation from the intended interventions [26, 29, 35–37, 39, 40, 43, 44, 51], both of which normally related to the lack of a pre-trial investigation and reporting plan. A common characteristic of studies that passed RoB screening with no concerns was studies that explicitly stated their aims and outcome measures prior to data collection and demonstrated adherence to their analysis plan [25, 27, 28, 31, 33, 41, 42, 46–48]. The selection of secondary outcomes with significant (or near-significant) differences between groups for reporting despite unclear clinical significance was a recurring concern [37, 39, 40, 45, 47, 48, 51].

**Table 2:** Risk of bias of included studies.




Low risk of bias is indicated by O. Some concern regarding bias is indicated by ~. High risk of bias is indicated by X. *LMWH* Low Molecular Weight Heparin, *VEGF* vascular endothelial growth factor.

### Primary prevention of PVR after RRD

A summary of findings of the included RCT for primary prevention of PVR after RRD is presented in Table 3. Asaria et al. [27]. found a beneficial effect of 5-Fluorouracil (5-FU), an antimetabolite that inhibits synthesis and fibroblast proliferation, and Low Molecular Weight Heparin (LMWH), an anticoagulant that binds many growth factors, in 87 patients in the intervention group, who had lower rates of post-operative PVR and better visual acuity after detachment surgery, but no differences in the rates of reoperation. However, these results were not replicated in larger studies conducted by Wickham et al. [46]. or Schaub et al. [41].

**Table 3:** Summary of findings in studies of primary prevention of proliferative vitreoretinopathy (PVR) after rhegmatogenous retinal detachment (RRD).

Intervention	Author	Intervention *n* =	Control *n* =	Primary outcome measured	Functional outcomes (Time; Actual Values; *P* Values; Summary) (Primary outcome in bold)	Anatomic outcomes (Time; Actual Values; *P* Values; Summary) (Primary outcome in bold)	Safety findings
Anti-Inflammatory							
Colchicine	Ahmadieh et al. [26]	76	85	BCVA	6 months **No raw BCVA data.** ***p*** = **0.41. No significant difference**.	6 months. PVR Grade C and Re-Detachment: Intervention: 11.8% (*n* = 9), Control: 15.3% (*n* = 13), *p* = 0.39. No significant difference in anatomical outcomes.	9.8% discontinued colchicine due to GI side-effects. Authors noted increased PVR in eyes treated with subsequent secondary pneumatic retinopexy. Pre-existing PVR most likely cause for failure of primary repair, hence unlikely causation.
Corticosteroids	Koerner et al. [37]	110	110	Anatomic reattachment rate at 6 months	6 months. LogMAR letters change: Intervention: −8, Control: −12. P = Not significant	6 months **Total reattachment rate: Intervention: 95.1% (*n*** = **97/102), Control: 89.4% (*n*** = **84/94). P** **=** **Not significant** Reattachment rate after first operation: Intervention: 86.4% (*n* = 95/110), Control: 85.5% (*n* = 94/110) p = Not significant Total number of re-operations: Intervention: 29, Control: 39. P = Not significant Reoperation PVR Stage B: Intervention: 20.7% (*n* = 6/29), Control: 53.8% *n* = 21/39. *P* ≤ 0.01 Cellophane appearance: Intervention: 19.8% (*n* = 20/101), Control: 39.1% (36/92). *P* ≤ 0.005 Cellophane including posterior pole: Intervention: 16.8% (*n* = 17/102), Control: 34.8% (*n* = 32/92) *p* ≤ 0.005 Cellophane only posterior pole: Intervention: 14.9% (*n* = 15/102), Control: 26.1% (*n* = 24/92) *p* = 0.05 Retina rigidity: Intervention: 5.9% (*n* = 6/102), Control: 13.8% (*n* = 13/94) p = Not significant Inner retinal wrinkling: Intervention: 6.9% (*n* = 7/102), Control: 14.9% (*n* = 14/94) *p* = 0.07 ERM: Intervention: 5% (*n* = 5/101), Control: 12.8% (*n* = 12/94). *p* = 0.05 PVR Grade A: Intervention: 1.0% (*n* = 1/102), Control: 4.3% (*n* = 4/94). p = Not significant PVR Grade B: Intervention: 22.5% (*n* = 23/102), Control: 45.7% (*n* = 43/94). *p* = 0.0005 PVR Grade C: Intervention: 2.0% (*n* = 2/102) Control: 3.2% (*n* = 3/94). p = Not significant Any Grade PVR: Intervention: 25.5% (*n* = 26/102), Control:53.2% (*n* = 50/94). *p* = <0.0002	No concerns
Anti-metabolite/Anti-proliferative							
5-FU and Heparin	Wickham et al. [46]	342	299	Retinal reattachment rate after 6 months without further operation	6 months. Visual acuity: No difference in Visual acuity outcome 0.53 (treatment), 0.48 (Control). *P* value 0.072. Significantly worse visual outcome for ‘macula on’ detachments in the treatment group. *P* value 0.0091	6 months. **Retina Reattachment Rate: Intervention group: 82.3% (*n*** = **269/327), Control: 86.8% (*n*** = **250/288).** ***P*** **value 0.12. No significant difference between groups**. IOP: Treatemnt: 16 (SD 4.36), Control: 16 (SD 4.00) Corneal clarity (clear): Treatment: 19.6% (*n* = 64/327), Control: 15.6% (*n* = 45/288)	**VA** worse for macula on detachments **IOP**: Treatment: 16 (SD 4.36), Control: 16 (SD 4.00)
5-FU and Heparin	Asaria et al. [27]	87	87	Development of PVR at 6 months	6 months. Change in visual acuity: no statistical difference in visual accuity outcome between the 2 groups. (however table states, placebo group had significantly worse final visual accuity. *P* = 0.048). Those that developed PVR had worse visual outcome *p* value = 0.0001	6 months. **Rate of developing PVR:** **Intervention: 12.6% (*n*** = **11/87), Placebo: 26.4% (*n*** = **23/87),** ***P*** **value** = **0.02. Statistically significant difference** Primary surgery success in retinal reattachment: Intervention: 78.2% (*n* = 68/87), Control 71.2% (*n* = 62/87), *P* = 0.47 Need for reoperations: Intervention: 19.5% (*n* = 17/87), Control 25.3% (*n* = 22/87), *P* = 0.19 reoperations resulting from developing PVR: Intervention: 10.3% (*n* = 9/87), Control 18.4% (*n* = 16/87)	No concerns
5-FU and Heparin	Schaub et al. [41]	163	162	Development of PVR grade CP1 or higher after 3 months	3 months Visual acuity: Intervention: 0.3 ± 0.4 (*n* = 134), Control: 0.3 ± 0.3 (*n* = 143), *P* = 0.443	3 months **Incidence of PVR:** **Intervention: 15/163 (9.2%), Control: 15/162 (9.1%),** ***p*** **value** = **0.47. No statistically significant difference** PVR grade CA: Intervention: 2% (*n* = 3/163) 36 participants not assessable, Control: 1% (*n* = 1/162) 32 participants not assessable. Additional operations to achieve retinal attachment: Intervention: 13% (*n* = 21/163), Control: 17% (*n* = 28/162) Drug adverse effects: Intervention: 6% (*n* = 10/163), Control: 7% (*n* = 11/162). *P* = 0.82	**Drug adverse effects**: Intervention: 6% (*n* = 10/163), Control: 7% (*n* = 11/162). *P* = 0.82
Growth factor modulation							
Anti-VEGF	Tousi et al [44]	12	15	BCVA	6 months: **LogMAR: 1.16(** ± **0.74) Control. 0.96(** ± **0.52) Intervention. No significant difference detected**.	6 months. Retina off: Intervention: 30% (*n* = 3/10), Control: 37.5% (*n* = 3/8). *p* = 0.99+ Retina on: Intervention: 70% (*n* = 7/10), Control: 62.5% (*n* = 5/8). *P* = 0.99. Nil significant difference in anatomical outcome.	No concerns.
Other							
Atropine and timolol	Tewari et al. [43]	Subgroup 2 (timolol) ‘Non-PVR group’ = 15 Subgroup 3 (timolol + atropine) ‘Non-PVR group’ = 15	Placebo (subgroup 1) ‘Non-PVR group’ = 15	Retinal reattachment after 3 months	No Data	3 months **Non- PVR group, retinal reattachment rates: Subgroup 1: 53.3% (*n*** = **8/15), Subgroup 2: 33.3% (*n*** = **5/15), Subgroup 3: 53.3% (*n*** = **8/15). No significant difference**.	No concerns

*BCVA* best-corrected visual acuity, *GI* gastrointestinal, *VA* visual acuity, *5-FU* 5-Fluorouracil, *LMWH* low molecular weight heparin.

### Primary prevention of PVR after OGI

A summary of findings of the included RCT for primary prevention of PVR after OGI is presented in Table 4. Guo et al. [36]. demonstrated promising results utilising intravitreal corticosteroids at the time of primary repair of OGI in 34 patients although both reviewers had concerns regarding the masking of surgeons (a common issue when using identifiable intravitreal interventions such as triamcinolone) as well as a high drop-out rate combined with the lack of a sensitivity analysis which undermined the analysis of results from a very heterogenous group of trauma patients [36]. No interventions demonstrated effectiveness without concerns of bias.

**Table 4:** Summary of findings in studies of primary prevention of proliferative vitreoretinopathy (PVR) after open globe injury.

Intervention	Author	Intervention *n* =	Control *n* =	Primary outcome measured	Functional outcomes (Time; Actual values; *P* Values; Summary) (Primary outcome in bold)	Anatomic outcomes (Time; Actual values; *P* Values; Summary) (Primary outcome in bold)	Safety Findings
Anti-Inflammatory							
Corticosteroids	Casswell et al. [31]	143	137	BCVA	6 months. **Intervention: 46.9% (*n*** = **61/130), Control: 43.4% (*n*** = **56/129).** ***p*** = **0.908**. **No signifcant difference in patients gaining 10 ETDRS letters in 6 months. Change in ETDRS BCVA at 6 months: Intervention: 19.4** ± **30.8, Control: 18.9** ± **29.2** ***p*** = **0.430**	6 months Retinal PVR re-detachment: Intervention: 33.9% (*n* = 42/124), Control: 28.2% (*n* = 35/124) *p* = 0.327 Stable complete retinal reattachment: Intervention: 51.6% (*n* = 65/126), Control: 64.2% (*n* = 79/123) *p* = 0.044 Stable macular retinal reattachment: Intervention: 54.0% (*n* = 68/126), Control: 66.7% (*n* = 82/123) *p* = 0.041 Tractional retinal detachment: Intervention: 28.2% (*n* = 35/124), Control: 24.4% (*n* = 30/123) *p* = 0.494 Hypotony (within 6 months): Intervention: 24.8% (31/125), Control: 22.6% (28/124) *p* = 0.680 Elevated IOP Intervention: 46.4% (58/125), Control: 31.5% (40/127) *p* = 0.016 Macular pucker: Intervention: 29.8% (37/124), Control: 20.5% (25/122) *p* = 0.093 Mean VFQ-25 at 6 months: Intervention: 72.0, Control: 71.9 *p* = 0.723	**Elevated IOP:** Intervention: 46.4% (58/125), Control: 31.5% (40/127) *p* = 0.016
Corticosteroids	Banerjee, Xing et al. [29]	20	20	Anatomic reapposition of the remaining retina to the retinal pigment epithelium in the absence of an internal tamponade agent at 6 months post primary vitrectomy surgery	6 months. Median ETDRS BCVA (IQR): Intervention: 31 (12.5–47.5), Control: 25 (0-65.0) VA change from baseline: Gain 10 letters Intervention: 80% (*n* = 16) Control: 52.6% (*n* = 10) Gain 20 letters Intervention:65% (*n* = 13) Control: 52.6% (*n* = 10) Gain 30 letters Intervention:50% (*n* = 10) Control: 42.1% (*n* = 8) Gain 40 letters Intervention: 45% (*n* = 9) Control: 31.6% (*n* = 6)	6 months. **Intervention: 50% (*n*** = **10/20), Control: 47.4% (9/19). Control [excluding early withdrawals]: 56.3% (9/16)**	**Elevated IOP**: Intervention: 35% (*n* = 7), Control 25% (*N* = 5)
Corticosteroids	Guo et al. [36]	34	34	Initial TPVR risk score vs PVR grading during vitrectomy	6 months BCVA (Log-MAR): Intervention: 1.70 ± 0.83, Control: 2.17 ± 0.83, *p* = 0.052 IOP (mm Hg): Intervention: 13.52 ± 4.21, Control: 14.26 ± 3.65, *p* = 0.413 VA change from baseline: *p* = 0.008 VA Improved: Intervention:92% (*n* = 23), Control: 63.64% (*n* = 14) VA No change: Intervention:0%, Control: 18.18% (*n* = 6) VA Deteriorated: Intervention: 8% (*n* = 2) Control: 9.09% (*n* = 2) NLP: Intervention: 8% (*n* = 2) Control: 36.36% (*n* = 8) VA of better than 0.01: Intervention: 48% (*n* = 12) Control: 22.73% (*n* = 5)	6 months **PVR Grade I: Intervention: 29.63% (*n*** = **8), Control: 12% (*n*** = **3)** **PVR Grade II: Intervention: 18.52% (*n*** = **5), Control: 24% (*n*** = **6)** **PVR Grade III: Intervention: 29.63% (*n*** = **8), Control: 8% (*n*** = **2)** **PVR Grade IV: Intervention: 22.22% (*n*** = **6), Control: 56% (*n*** = **14)** ***p*** = **0.028** **Initial injury TPVR score in the TA group was significantly higher than that in the control group Intervention: 9.30** ± **0.82, Control: 6.44** ± **1.06** ***p*** = **0.036** Retina attached: Intervention:88% (*n* = 22), Control: 63.64% (*n* = 14) Retina detached: Intervention: 12% (*n* = 3) Control: 36.36% (*n* = 8) Retinal attachment rate *p* = 0.049 Macula attached: Intervention: 24% (*n* = 6) Control: 40.91% (*n* = 9) Macula not attached: Intervention: 76% (*n* = 19), 59.09% (*n* = 13) Macula attachment rate *p* = 0.215 PVR recurrence: Intervention: 40% (*n* = 10), Control: 59.09% (*n* = 13) No PVR Recurrence: Intervention: 60% (*n* = 15), Control:40.91% (*n* = 9) PVR Recurrence rate *p* = 0.191	**IOP (mm Hg)**: Intervention: 13.52 ± 4.21, Control: 14.26 ± 3.65, *p* = 0.413

*BCVA* best-corrected visual acuity, *ETDRS* early treatment of diabetic retinopathy, IOP intraocular pressure, *IQR* interquartile range, *TA* triamcinolone, *TPVR* traumatic proliferative vitreoretinopathy, *VA* visual acuity.

### Treatment of established PVR

#### Corticosteroids

A summary of findings of the included RCT for secondary treatment of PVR is presented in Table 5 [53]. A study investigating the efficacy of slow-release 0.7 mg dexamethasone intravitreal implant as a secondary preventative adjunct for PVR grade C treatment found less cystoid macular oedema at 6 months post-op, but no difference in the anatomic or functional success compared to vitrectomy with SiO placement without the dexamethasone implant [28].

**Table 5:** Summary of findings in studies of secondary treatment of proliferative vitreoretinopathy (PVR) after rhegmatogenous retinal detachment (RRD).

Intervention	Author	Intervention *n* =	Control *n* =	Primary outcome measured	Functional outcomes (Time; Results; *P* Values; Primary outcome in bold)	Anatomic outcomes (Time; Results; *P* Values; Primary Outcome in bold)	Safety findings
Anti-Inflammatory							
Acetyl-salicylic acid	Kralinger et al. [38]	15	14	Incidence of retinal redetachment at 6 months requiring reoperation	6 months Visual acuity: no significant different between the 2 groups. *P* value = 0.913	6 months **Redetachment rate: Intervention: 33.3% (*n*** = **5/15), Control: 7% (*n*** = **1/14).** ***P*** > **0.169. not statistically significant**. note. This information was not clear and was misleading in the abstract, which states ‘redetachment rate was the same for both groups.’ Adverse events: Intervention: 0% (*n* = 0/15), Control: 14% (*n* = 2/14). 1 corneal ulcer requiring keratoplasty, 1 large vitreous haemorrhage. Statement of demomstration of safety of intervention AS SiO based on visual acuity results and clinical examination.	Adverse events in control group only, not attributable to intervention
Corticosteroids	Ahmadieh, Feghhi et al. [25]	38	37	Retinal reattachment rate at 6 months post-surgery without need for reoperation	6 months Visual acuity: Intervention: −0.9 ± 0.9, Control −0.9 ± 0.8, *p* = 0.74	6 months. **Retinal reattachment without reoperation: 82.4% (*n*** = **32) Treatment group. 78.4% (*n*** = **29) Control.** ***p*** = **0.5. No difference in retinal reattachment rate** Rate of recurrent PVR: Intervention: 28.9% (*n* = 11), Control: 29.7% (*n* = 11), *p* = 0.94 Reoperation rate: Intervention: 15.8% (*n* = 6), Treatment: 21.6% (*n* = 8), *p* = 0.52 Intraocular pressure change: Intervention: 5.6 ± 6.8 mmHg, Control: 4.6 ± 6.8. *p* = 0.59 Nil significant difference detected in any outcome.	No concerns.
Corticosteroids	Trenado-Luna et al. [45]	18	20	Retinal reattachment at 3 months	3 months. Pre-op mean Logmar VA: Intervention: 1.93 ± 0.50, Control: 2.01 ± 0.50. Post-op mean Logmar VA: Intervention: 1.36 ± 0.71, Control: 1.86 ± 0.82 ‘significant difference betwee the final VA of patients in both groups’ P = 0.048	3 months **Retinal attachment: intervention: 61.1 (*n*** = **11), Control 20% (*n*** = **4),** ***P*** = **0.013** Post-op PVR: No PVR: Intervention: 44.4% (*n* = 8), Control: 20% (*n* = 4) Grade A PVR: Intervention: 0, Control: 5% (*n* = 1) Grade B PVR: Intervention: 22.2% (*n* = 4), Control:10% (*n* = 2) Grade C PVR: Intervention: 33.3% (*n* = 6), Control: 65% (*n* = 13)	Two patients removed from treatment group due to autoimmune conditions identified during study
Corticosteroids	Banerjee et al. [28]	70	70	Stable retinal reattachment rate post oil removal without further surgery	6 months Mean ETDRS letters BCVA at 6 months: Intervention: 38.3, Control: 40.2. Mean change in ETDRS from baseline: Intervention: 24.5, Control: 23.1. Eyes achieving >55 ETDRS letters: Intervention: 30% (*n* = 21), Control 24% (*n* = 17).	6 months. **Stable retinal reattachment rate post oil removal without further surgery intervention: 49.3% (*n*** = **34), control: 46.3 (*n*** = **32).** ***p*** = **0.733. No significant difference**. PVR Recurrence: Intervention: 57% (*n* = 40), Control: 59% (*n* = 41). Complete Retinal Reattachment: Intervention: 53.6% (*n* = 37), Control: 62.3% (*n* = 43). Stable Posterior Reattachment: Intervention: 66.7% (*n* = 46), Control: 62.3% (*n* = 43). Tractional Retinal Detachment: Intervention: 22% (*n* = 15) Control: 19% (*n* = 13). Further procedures to achieve reattachment: 1 Further procedure: intervention: 36.2% (*n* = 25), Control: 30.4% (*n* = 21). 2 Further Procedures: Intervention: 4.4% (*n* = 3), Control: 16% (*n* = 11). CMO Present: Intervention: 42.7% (*n* = 29), Control: 67.2% (*n* = 45), *p* = 0.004. Foveal Thickness > 300 microns: Intervention: 47.6% (*n* = 30), Control: 67.7% (*n* = 42), *p* = 0.023. ERM/Macular Pucker: Intervention: 57% (*n* = 40), Control: 58.6% (*n* = 41). ERM Surgery: Intervention: 47% (*n* = 33), Control: 44.3% (*n* = 31). IOP Variation: No significant difference. Overall: Difference noted in >1 surgeries rate, CMO presence.	Higher number of adverse events in control group
Corticosteroids	Dehghan et al. [34]	25	27	BCVA	6 months **Mean postoperative visual acuity: intervention: 0.62** ± **0.39 logMAR, Control: 0.78** ± **0.58 logMAR.** ***p*** = **0.39**. **Difference between preoperative and postoperative visual acuity: Intervention: 0.85** ± **0.62 logMAR, Control: 0.65** ± **0.61 logMAR.** ***p*** = **0.36**.	Variable times Choroidal detachment (within 1 week): intervention: 16% (*n* = 4), Control: 11.1% (*n* = 3). *p* = 0.45 PVR (within 6 months) Intervention:4% (*n* = 1), Control: 11.1% (*n* = 3). *p* = 0.33 Macular oedema (within 6 weeks): Intervention: 12% (*n* = 3), Control: 18.5% (*n* = 5). *p* = 0.39 No difference between intervention and control.	No concerns
Anti-metabolite/Anti-proliferative							
Daunorubicin	Kumar et al. [39]	15	15	Complete retinal reattachment after 3 months	3 months. BCVA: >6/60, 3/60–6/60, <3/60 Intervention: 20% (*n* = 3), 40% (*n* = 6), 40% (*n* = 6) Control: 6.6% (*n* = 1), 26.6% (*n* = 4), 66.6% (*n* = 10) *p* = Not significant	3 months **Complete retinal reattachment: Intervention: 86.7% (*n*** = **13), Control: 10 eyes (66.7%). p** **=** **Not significant** Reduction in Vitreous Haze in Intervention vs Control: 1 week: *p* = <0.0001, 1 month: *p* = 0.0022, 3 months: *p* = 0.0044	No concerns
Daunorubicin	Wiedemann et al. [47]	145	141	Treatment success after 6 months, defined as complete reattachment within the cerclage without vitreoretinal reoperation	6 months. VA Improved: Intervention: 83.1% (*n* = 113) Control: 76.6% (*n* = 98) VA Unchanged: Intervention: 12.5% (*n* = 17), Control: 19.5% (*n* = 25) VA Deteriorated: Intervention: 4.4% (*n* = 6), Control: 3.9% (*n* = 5)	6 months **Complete reattachment: Intervention: 62.7% (*n*** = **89/142) (CI 54.2%–70.6%), Control: 54.1% (*n*** = **73/135) (CI 45.3%–62.7%)** ***P*** = **0.07** Overall attachment rates with or without reoperations (6 months): Intervention: 76.5% (104/136), Control: 78.0% (99/127) Overall attachment rates with or without reoperations (12 Months): Intervention: 80.2% (105/131), Control: 81.8% (103/126) Number of reoperations within 1 year: None:Intervention: (*n* = 95) Control: (*n* = 76) 1:Intervention: (*n* = 42) Control: (*n* = 44) 2: Intervention: (n = 4) Control: (*n* = 16) 3: Intervention: (*n* = 4) Control: (*n* = 4) 4: Intervention: (*n* = 0) Control: (*n* = 1)	No concerns
DNA–RNA chimeric ribozymes	Schiff et al. [42]	High dose: 60, Low Dose: 58, Total: 118	57	Failure rate of retinal reattachment (a) due to PVR and (b) due to other causes	24 weeks BCVA: Absolute values not reported. *p* = 0.64, No significant difference between all groups.	24 weeks **Retinal detachment:** **PVR: Intervention High: 33% (*n*** = **18), Intervention Low: 24% (*n*** = **13), Control: 21% (*n*** = **10),** ***p*** = **0.37** **All causes: Intervention High: 38% (*n*** = **21), Intervention Low: 40% (*n*** = **22), Control: 34% (*n*** = **16),** ***p*** = **0.83** **Reproliferation without detachment Intervention High: 63% (*n*** = **21), Intervention Low: 53% (*n*** = **17), Control: 53% (*n*** = **16),** ***p*** = **0.62**	No concerns
5FU and Heparin	Charteris et al. [33]	73	84	Posterior retinal reattachment after removal of silicone oil without any reoperations at 6 months	No Data	6 months **Retinal Reattachment Rate: intervention 39 (56%), control 40 (51%),** ***X***^2^ = **0.29,** ***P*** = **0.589. no significant difference between groups** Full Retina reattachment at 12 months (100 cases); Intervention: 85% (*n* = 40/47), control: 85% (*n* = 45/53) Localised anterior tractional retinal detachment at 12 months; Intervention: 6% (*n* = 3/47), control: 4% (*n* = 2/53) Posterior tractional retinal detachment at 12 months; Intervention: 4% (*n* = 2/47), control: 4% (*n* = 2/53) Rhegmatogenous retinal detachment at 12 months; Intervention: 4% (*n* = 2/47) (0), control: 7% (*n* = 4/53) (2). *P* = 0.768 Fully attached macular (no pucker) at 12 months (99 cases): Intervention: 79% (*n* = 34/47), control: 69% (*n* = 36/52) Puckered macular at 12 months: Intervention: 15% (*n* = 7/47), control: 27% (*n* = 14/52) Detached (tractional) macular at 12 months: Intervention: 9% (*n* = 4/47) (2), control: 4% (n = 2/52) (0) Macular hole at 12 months: Intervention: 2% (*n* = 1/47), control: 0% (*n* = 0/52) Cystoid macular oedema at 12 months: Intervention: 2% (*n* = 1/47), control: 0% (*n* = 0/52). *P* = 0.261 Complications (98 cases): Glaucoma; Intervention: 0, Control: 3 Hypotony; Intervention: 9, Control: 7 Keratopathy; Intervention: 5, Control: 2 Cataract extraction; Intervention: 21, Control: 29. non-significance	**Complications** (98 cases): Glaucoma; Intervention: 0, Control: 3 Hypotony; Intervention: 9, Control: 7 Keratopathy; Intervention: 5, Control: 2 Cataract extraction; Intervention: 21, Control: 29. No significant difference
5FU and Heparin	Nasr et al. [50]	21	21	Rate of recurrent RD due to PVR formation within 12 weeks	12 weeks BCVA > CF: intervention: 4/19 (20%), Control: 2/17 *p* = 0.035	12 weeks **Recurrent** **RD at 12 weeks intervention: 4/21 (19%). Control: 7/21 (33%)** ***p*** = **0.292** Any grade or degree of PVR at 6 weeks: Intervention: 8/20 (40%), Control: 8/20 (40%) Any grade or degree of PVR at 12 weeks: Intervention: 7/20 (35%), Control: 8/20 (40%) *p* = 0.038 Secondary Procedures at 12 weeks: Intervention: 7/20 (35%), Control: 5/19 (21%) *p* = 0.263	No differences in adverse outcomes between groups, intervention group required later secondary interventions for post op complications (statistically significant)
5FU and heparin	Ganekal et al. [35]	20	20	Posterior retinal attachment rate at 6 months	6 months Visual outcomes of 20/800 or better: Intervention: 45% (*n* = 11/20), Control: 30% (*n* = 6/20), *P* > 0.05	6 months **Retinal Attachment rate: intervention: 65% (*n*** = **13/20), control: 60% (*n*** = **12/20).** ***P*** **value 0.744. No significant difference in outcome between groups**. Postoperative PVR: Intervention: 45% (*n* = 9/20), Control: 55% (*n* = 11/20), *P* value 0.527 Complication rate: statement of no significant difference in complication rate or drug toxicity between groups, but numerical data is not clearly presented.	No side effects from intervention drugs
Methotrexate	Rajan et al. [51]	21	20	Anatomical success (fully attached, partial attached mac on, partial attached mac off)	6 months. BCVA post PPV was 0.674 ± 0.615 (LogMAR) in case group and 0.219 ± 0.828 in control group (*p* = 1)	6 months **Fully attached Intervention:15, Control:9** ***p*** = **0.086** **Partial detachment macula on Intervention:6 Control:4** ***p*** = **0.522** **Partial detachment macula off Intervention:0 Control:7** ***p*** = **0.003** Post oil removal, 5/21 (3 intervention, 2 control) fully attached retinas progressed to mac off.	None
300 Gy radiation	Binder et al. [30]	30	30	Retinal reattachment after 6 months	6 months Visual acuity: No apparent difference between the groups, no statistical analysis done	6 months. **Retinal reattachment:** **Intervention: 63% (*n*** = **19/30), Control: 57% (*n*** = **17/30). P value** ***P*** = **0.479. No significant difference between groups. Retinal reattachment at 14–36 months: Intervention: 50% (*n*** = **15/30), Control: 57% (*n*** = **17/30).** ***P*** = **0.473** Irradiation appears to delay the onset of proliferation by 7–14 months but does not ultimately affect the reattachment or visual result	No side effects of radiation observed after 3 years
Growth factor modulation							
Heparin	Williams et al. [48]	25	34	Rate of RRD resulting from reproliferation and requiring vitrectomy revision	6 months Visual acuity: no significant difference in visual acuity between the 2 groups	6 months **58 eyes have follow-up data to 6 months** **Redettachment rate: Intervention: 16% (*n*** = **4/25), control: 26.5% (*n*** = **9/34).** ***P*** = **0.26. No significant difference between groups** Retinal reattachment rate after 1 operation: Intervention:80% (*n* = 20/25), Control: 65% (*n* = 22/34). *P* = 0.16. Not significant. Postoperative haemorrhage: Intervention: 20% (*n* = 5/25), Control: 0% (*n* = 0/34). *P* = 0.01. Significant hypotony (both with attached and detached retinas): Intervention: 4% (*n* = 1/25), Control: 21% (*n* = 7/33). *P* = 0.063. Not significant Redetachment rate in subgroup requiring retinotomy (17 eyes): Intervention: 0% (*n* = 0/8), Control: 56% (*n* = 5/9). *P* = 0.02. significant	**Postoperative haemorrhage**: Intervention: 20% (*n* = 5/25), Control: 0% (*n* = 0/34). *P* = 0.01. Significant. No related difference in visual outcome
Heparin	Kumar, Nainiwal et al. [40]	15	15	Post operative fibrin (media clarity) at 3 months as measured by Nussenblatt classification	3 months Visual acuity: No statistical difference in visual outcome *P* = 0.376	3 months **Table of raw values presented showing media clarity score at 1 week, 1 month and 3 months follow-up** **Values at 3 month follow-up presented below:** **Intervention: 4** + = **0% (0/15), 3** + = **0% (0/15), 2** + = **7% (1/15), 1** + = **13% (2/15), 0** = **80% (12/15)** **Control: 4** + = **0% (0/15), 3** + = **0% (0/15), 2** + = **60% (9/15), 1** + = **33% (5/15), 0** = **7% (1/15)** ***P*** = **0.0042** Retinal Attachment rate: no data presented. A statement of 50% better chance of reattachment in Study group. Re-detachment rate noted upon: Intervention: 7% (*n* = 1/15), Control: 27% (*n* = 4/15). no statistical analysis Intraoperative bleeding: Intervention: 33% (*n* = 5/15), Control: 20% (*n* = 3/15). no statistically significant difference	**Intraoperative bleeding**: Intervention: 33% (*n* = 5/15), Control: 20% (*n* = 3/15). No significant difference
Other							
Infliximab	Younes et al. [52]	30	30	Anatomical success (complete reattachment 6 months post oil removal)	9 months Final logMAR BCVA, (mean ± SD (Snellen Equivalent)), Intervention: 0.96 ± 0.4 (20/180), Control: 1.14 ± 0.4 (20/280) *p* = 0.044	9 months (6 months post oil removal) **Single operation success: Intervention: 26 (86.7%) Control: 23 (76.7%)** ***p*** = **0.317.** **Final anatomical success: Intervention: 30 (100%), Control: 29 (96.7%)** ***p*** = **1** Number of surgeries to achieve retinal attachment (Mean ± SD) Intervention: 1 ± 0.1 Control: 1 ± 1.1 *p* = 0.482 Median (range) 1 Intervention: 1 (1–2) Control: 1 (1–3) Time to recurrence (weeks) Mean ± SD Intervention: 4 ± 1 Control: 4 ± 1 *p* = 1.000 Median (range) Intervention:4 (3–5) Control:4 (2–5) Time of SOR (months) Mean ± SD Intervention:3.9 ± 0.7, Control:4 ± 0.7 *p* = 0.745 Median (range) 4 (3–6) 4 (3–6) 4 (3–6) Final IOP mean ± SD (mmHg) Intervention:16 ± 4 Control:16 ± 3 *p* = 0.773 Central Macula Thickness mean ± SD (μm) Intervention:213 ± 33, Control: 212 ± 39 *p* = 0.585 ERM formation, n (%) Intervention: 3 (10%) Control: 5 (16.7%) *p* = 0.706	No concerns
Atropine and timolol	Tewari et al. [43]	**Subgroup 2** (timolol) ‘PVR group’ = 15 **Subgroup 3** (timolol + atropine) ‘PVR group’ = 15	Placebo (**subgroup 1**) ‘PVR group’ = 15	Retinal reattachment after 3 months	No Data	3 months Retinal reattachment Rates: 1 Week: PVR Group: Sub 1: 0% (*n* = 0/15), Sub 2: 0% (*n* = 0/15), Sub 3: 0% (*n* = 0/15). Not significant 1 Month: PVR Group: Sub 1: 6.6% (*n* = 1/15), Sub 2: 6.6% (*n* = 1/15), Sub 3: 0% (*n* = 0/15). Not significant 2 Months: PVR Group: same results as month 1 **3 Months: PVR Group: same as month 1**. Statement made that no statistical difference in retinal reattachment rate between PVR and Non-PVR groups. No P value given.	No concerns
Retinoic acid	Chang et al. [32]	16	19	Retinal attachment rate at 1 year	1 year Ambulatory VA: Intervention: 56.3% (*n* = 9/16), control: 10.5% (*n* = 2/19), *P* = 0.009. Significantly better VA in intervention group.	1 year **Initial reattachment rate (no repeat surgeries): Intervention: 87.5% (*n*** = **14/16), Control: 52.6% (*n*** = **10/19).** ***P*** = **0.027. Significant difference**. **reattachment rate (repeat surgeries allowed): Intervention: 93.8% (*n*** = **15/16), Control: 63.2% (*n*** = **12/19).** ***P*** = **0.047. Significant difference** Intervention group had 2x repeated surgeries, Control group had 10x repeat surgeries. Macular pucker rate: Intervention: 18.8% (*n* = 3/16), Control: 78.9% (*n* = 15/19) *P* = 0.001. significantly reduced Macular pucker in the intervention group. RA related Side effects: Dry mouth: 68.7% (*n* = 11/16), Dryness and peeling of skin: 56.3% (*n* = 9/16), skin itching: 31.3% (*n* = 5/16), Joint and muscle pain: 6.3% (*n* = 1/16), headache: 6.3% (*n* = 1/16), hypercholesterolaemia: 6.3% (*n* = 1/16).	Normal side effects associated with Retinoic Acid (dry mouth, dryness and peeling of skin, skin itching, muscle pain, headache, and hypercholesterolaemia) only.

*BCVA* best-corrected visual acuity, *CMO* cystoid macular oedema, *ERM* epiretinal membrane, *GI* gastrointestinal, *IOP* intraocular pressure, *VA* visual acuity, *5-FU* 5-Fluorouracil, *LMWH* low molecular weight heparin, *RA* retinoic acid.

Trenado-Luna at al. studied intravitreal dexamethasone implant in 18 patients with grade B or higher PVR, finding greater reattachment rates and better visual acuity in patients treated with dexamethasone implant at the time of vitrectomy, but had several issues concerning the randomisation and masking processes which may have led to the small patient numbers (with significant differences between control and test group demographics) giving a falsely significant result [45].

#### Antimetabolites and heparin

A combination therapy of steroids or the anti-metabolite 5-FU with LMWH was also studied in 73 patients, which did not reveal any improvement in anatomical or visual outcomes in macular-involving PVR detachments and resulted in worse visual outcomes for patients with macula-sparing PVR retinal detachments [46]. Williams et al. [48]. studied patients with grade C3 or D PVR, finding that intraoperative heparin and dexamethasone in the infusion fluid of 25 patients had no effect on retinal reattachment rate or visual acuity, but was associated with increased rates of post-operative vitreous haemorrhage (20% vs 0%; *p* = 0.01) and improved (lower) rates of the secondary outcomes of patients requiring retinotomy. Nasr et al. [50]. examined paediatric populations with complex detachment and established PVR, noting slightly improved visual acuity with 5-FU and LMWH but no significant difference in anatomical outcomes. Kumar et al. [40]. studied intraoperative LMWH 15 patients with grade D1-3 PVR, finding improved media clarity (vitreous haze), a subjective grading scale outcome of doubtful clinical significance in a probably unmasked assessor, while demonstrating no significant benefit in objectively measurable outcomes [40].

Repeated intravitreal Methotrexate injection was used by Rajan et al. [51] to prevent recurrent RRD in patients with established PVR Grade C, reporting no difference in overall redetachment rate or BCVA, but a significant difference in favour of treatment in the rate of macula on vs macula off detachments under oil. However, RoB reviewers were concerned that anatomical outcomes were assessed by same unmasked assessors who also decided timing of oil removal. Younes et al. [52]. assessed intravitreal infliximab before silicone oil insertion, demonstrating no difference in final anatomical success or single operation success rate 6 months after oil removal, but did find a modest improvement in final BCVA in favour of the intervention group.

The Daunorubicin Study Group investigated the safety and efficacy of daunorubicin, an anthracycline antibiotic that arrests cell proliferation and cell migration, during vitrectomy in 145 eyes with grade C2 or higher PVR. They found that daunorubicin use resulted in a small reduction in the reoperation rate in PVR patients undergoing retinal surgery. There was no difference in visual acuity and reattachment rate at one year [47]. There were some concerns with potential for bias regarding the reoperation rate, as it was unclear how many analyses were performed and limited baseline data were available.

A small RCT utilising prolonged dosing of oral retinoic acid [32] showed positive results in both retinal reattachment rates and visual acuity in 16 patients with primary RRD associated with established PVR.

## Discussion

This systematic review found a lack of high-quality evidence to support any one of the wide variety of potential non-surgical interventions aiming to improve the outcomes of patients with OGI or RRD with respect to PVR. To date, 27 RCTs, including 3375 patients, have examined the efficacy and safety of non-surgical primary and secondary interventions for PVR after OGI and RRD, examining a range of interventions, including corticosteroids, anti-metabolites, anti-VEGF, heparin, infliximab, methotrexate, and retinoic acid, without clear benefit compared to control groups. Potentially positive results were reported in two small studies: of intravitreal triamcinolone at the time of primary repair [36], and of oral retinoic acid in secondary treatment of established PVR [32], although these results require confirmation.

The varied and relatively uncommon nature of OGI in post-industrial nations, combined with decentralised ophthalmic trauma services, means that individual surgeons deal with post-OGI PVR rarely and may be unfamiliar with managing primary OGI and subsequent PVR. A relative paucity of cases means that many trauma studies included relatively few patients. In contrast, RRD is common, but the relatively low rate of PVR after RRD (compared to OGI) means that studies of primary prevention must be very large to achieve adequate statistical power. Studies of secondary treatment may require fewer patients, but the challenge facing candidate therapies is to reverse an established disease process, which is potentially more difficult than preventing its occurrence in the first place.

Despite positive results in pre-clinical models, for instance, of corticosteroid treatment and anti-metabolites [54], all therapies have failed to show efficacy in large, well-designed prospective human clinical trials, such as those reported by Wickham et al. [46] or Schaub et al. [41] of 5-FU, or Wiedemann et al. [47] of daunorubicin, or Banerjee et al. [28] and Casswell et al. [31] of corticosteroids.

Corticosteroid therapy has been the most investigated adjunct and remains the most commonly used in clinical practice. Clinical trials evaluating intravitreal triamcinolone acetonide showed a poor efficacy in treating established PVR [25, 55]. A RCT of intravitreal triamcinolone acetonide injection at the time of pars plana vitrectomy with SiO tamponade for secondary treatment in grade C PVR showed no improvement in anatomic success, visual acuity, or PVR development [55]. Triamcinolone acetonide is sometimes employed in retinal detachment repair for vitreous visualisation, but a large prospective multicentre study showed no difference in outcomes between patient undergoing vitrectomy with triamcinolone assisted visualisation and those without (although a confounding factor in this case may be that at the conclusion of vitrectomy there may be minimal amounts of triamcinolone remaining in the vitreous cavity) [25]. A 1-year RCT examining patient outcomes after different vitreoretinal surgeries found that intraoperative use of triamcinolone did not improve visual outcomes in the RRD cohort or reduce the rate of post-operative retinal detachment after vitrectomy [49]. Subconjunctival corticosteroid followed by extended courses of topical corticosteroid is routine after most vitreoretinal surgeries, especially in cases of trauma or cases thought to be high risk for PVR, which may confound the findings of studies and alter the impact of any intervention. There is therefore no evidence that corticosteroid therapy in any form reduces the occurrence of PVR, but it may reduce cystoid macular oedema [28], and is commonly used to manage inflammation.

Guo et al. reported positive findings utilising intravitreal corticosteroids at the time of primary repair (which is distinct from the negative ASCOT findings, where corticosteroids were given at the time of vitrectomy) [31], representing an intervention as early as possible in the PVR development cycle [36]. If these findings could be replicated in larger studies, they may represent a potential, simple, effective intervention. Crucially, post-operative endophthalmitis rates were not increased in patients receiving intravitreal steroids at the time of primary repair, which may be one argument against intravitreal corticosteroids in this cohort. All trials investigating OGI suffer from similar problems of relatively low patient numbers and heterogeneity of injury, which further complicates detailed analysis and comparison [31, 36].

One retrospective study evaluating patients with severe recurrent PVR and tractional retinal detachment or severe intraocular inflammation at high risk for PVR found that these patients had a lower incidence of PVR when treated with intravitreal methotrexate infusion during vitrectomy [56], and Rajan et al. [51]. reported positive subgroup analysis results in a small trial of methotrexate in patients with established PVR. While RoB assessment raised concerns with these studies, the large, multicentre GUARD trial of intravitreal Methotrexate (ADX-2191) is ongoing and results are awaited [57].

A small (*n* = 35) RCT of oral retinoic acid use in patients with PVR grade C undergoing vitrectomy demonstrated significantly lower rates of retinal re-detachment, macular pucker formation and improved vision in patients being treated with oral retinoic acid [32]. However, a larger prospective cohort study (DELIVER study) found that Isotretinoin may reduce the rate of PVR-related re-detachment in high-risk patients but did not alter the course of disease in patients with established PVR [58]. The positive results of the initial small study do not, therefore, seem to have been replicated. However, some variance between the two studies may warrant a larger investigation to attempt to more closely replicate the original RCT with more patients.

Safety concerns attributable to the intervention were noted in three studies: Wickham et al. [46], who reported worse visual outcomes after 5-FU treatment, and Williams et al. [48], who reported greater rates of post-operative vitreous haemorrhage with heparin treatment. Casswell et al. noted an increased frequency of raised IOP in the corticosteroid group, which is a well-recognised side effect [31].

Limitations of this review include the small number of RCTs included per intervention; for example, only corticosteroids and 5-FU with Heparin had more than two RCTs, although overall, our review included a large number of patients (*n* = 3255). A greater number of studies of each intervention may be available were non-RCT designs were included, but when RCTs are possible and available, treatment decisions should not be based on non-RCT data. Not all studies that examined interventions in conjunction with vitrectomy specified the extent to which vitrectomy was performed (core vs peripheral vs shave of vitreous base), which may also introduce surgical variation into the comparison of non-surgical interventions.

## Conclusion

This review did not identify any evidence of a proven effective non-surgical intervention for the treatment or prophylaxis of PVR. At present, the current techniques of surgical intervention for established PVR remain the only effective option.

Until an effective preventive strategy with minimal adverse effects is identified, the use of potential pharmacological agents for primary prevention of PVR is best targeted at high-risk patients to increase the power of studies to detect an effect. Identification of risk factors for PVR, and detailed assessment, such as utilising the revised Retina Society Classification, is therefore crucial for developing a pharmacologic prophylaxis.

Future investigation may be warranted of intravitreal steroids at the time of primary globe repair of OGI and for prolonged (>8 weeks) oral retinoid administration in patients with established PVR, as both interventions demonstrated potentially positive results in well-designed small studies. There may be benefits to trialling Methotrexate and Infliximab in cases of RRD where PVR has not yet established itself or become severe in order to better assess their efficacy.
